# Cassava starch-based hot melt adhesive for textile industries

**DOI:** 10.1038/s41598-024-70268-y

**Published:** 2024-09-09

**Authors:** Asmare Tezera Admase, Desalegn Abera Mersha, Addisu Yenesew Kebede

**Affiliations:** 1 Department of Chemical Engineering, Faculty of Chemical and Food Engineering, Bahir Dar Institute of Technology, Bahir Dar University, Bahir Dar, Ethiopia; 2 Department of Mechanical Engineering, Faculty of Mechanical and Industrial Engineering, Bahir Dar Institute of Technology, Bahir Dar University, Bahir Dar, Ethiopia

**Keywords:** Polyester cotton fabric, Hot melt, Tannic acid, Kaolin clay, Cassava starch, Chemical modification, Chemical engineering

## Abstract

The textile industry uses a lot of adhesives to join materials together, and many of these adhesives use petroleum-based ingredients that are harmful to the environment. To replace petroleum-based adhesives with a more environmentally friendly option for the textile industry, this study set out to create and evaluate a hot-melt adhesive derived from cassava starch. By adding kaolin clay as a filler and tannin as a tackifier in different ratios of starch, the created adhesive was enhanced. Tannic acid to starch ratios of 2:1, 6:1, and 10:1 w/w and kaolin to starch ratios of 3:1, 5:1, and 7:1 w/w were used to investigate the effects of clay and tackifier, respectively. The adhesives’s viscosity, moisture content, tensile strength, and shear strength were then measured. The presence of kaolin and tannic acid in starch-based adhesives favored a good interaction between the adhesive’s ingredients. The adhesive’s maximum shear strength was measured at 4.93 ± 0.11 Mpa when dry and 0.263 ± 0.21 Mpa when wet. The current data indicate that the optimal tensile strength was determined to be 3.45 ± 0.22 MPa. This result showed that hot melt adhesives based on cassava starch would be a good environmentally friendly substitute for petroleum-based adhesives, and more study in this field is necessary.

## Introduction

Adhesives have broad applications across industries such as food processing, pharmaceuticals, confectionery, beverages, pulp and paper, chemicals, cosmetics, binders and adhesives, packaging and printing, fermentation, and textiles^1,2^. Presently, the majority of adhesives utilized in industrial settings to bond wood panels are synthetic in nature, namely phenol–formaldehyde, urea–formaldehyde, and melamine–formaldehyde. However, these synthetic adhesives carry inherent risks to human health and contribute to air pollution due to the release of carcinogenic gases during the production and application of wood-based panels^3^. In recent years, there has been growing industrial and research interest in bio-based wood adhesives as they offer an environmentally friendly and renewable alternative to conventional petroleum-based synthetic adhesives used in the wood-based industry^4^.

Biomass-based adhesive materials are becoming increasingly important as sustainable and eco-friendly alternatives. Starch based adhesives derived from renewable biomass sources offer a viable option to traditional adhesives (which are often petroleum-based and environmentally impactful)^5^. By utilizing biomass as a feedstock, these adhesives help reduce reliance on non-renewable resources and mitigate environmental consequences^5,6^. Starch, a polysaccharide abundantly present in the roots, seeds, and stalks of staple crops such as wheat, rice, potato, corn, and others. It is an organic polymer and a naturally occurring substance that possesses several advantageous characteristics such as biodegradability, affordability, and renewability^7,8^.

Cassava has a high starch concentration compared to other starch sources, and it has been studied for use in the production of bio-based adhesives^9–12^. Because it can create a clear paste, has a low gel formation temperature, is stable after the gel is formed, and has great film characteristics, cassava starch is a more advantageous raw material for bio-adhesives than other starches^13^. Cassava starch can easily be gelatinized to produce homogenous adhesives because of the low gelatinization temperature. However, the water resistance of the cassava starch-based adhesive is usually diminished due to the presence of hydroxyl with a type of hydrogen bond in starch molecules^14,15^. Therefore, to increase the resultant product’s water resistance, steps must be taken to reduce the quantity of hydroxyl groups in cassava starch molecules.

To optimize the performance of starch as a wood adhesive, it is crucial to chemically modify or crosslink its molecular structure in conjunction with fillers, thereby enhancing the efficiency and effectiveness of starch-based wood adhesives^16–18^. To overcome limitations like poor water resistance and slow drying, starch is modified through physical methods (microwave, ultrasonic irradiation, reinforced with fillers) and chemical methods (esterification, oxidation, etherification) to enhance its performance^19–21^. There are a number of detailed literatures on the use of tannins for wood adhesives^5,12,22,23^. Based on their presence in various parts of plants such as bark, wood, leaves, seeds, roots, and even the plant galls are the major sources of tannin extractions used for various purposes^24–27^. Tannic acid contains glucose linking through ester bonds to an average of nine to ten molecule of gallic acid. Several research studies have shown that tannins may be used successfully as corrosion inhibitors, as a tackifiers thus they have been proposed for anticorrosive paints and pretreatment solutions^28^. Tannin-starch composite^29^ is an environmentally friendly solution with no formaldehyde emissions that may also be utilized for wood and wood composite adhesives. In the process of making plywood, Moubarik et al.^30^ reported partially substituting a resin based on corn starch and quebarcho tannin for phenol–formaldehyde. It was discovered that 20% that is, 15% cornstarch and 5% quebracho tannin—was the ideal replacement value. This resin’s inclusion increased the water resistance and decreased formaldehyde emissions. For internal plywood, the same authors created a cornstarch-tannin adhesive that is non-toxic and non-volatile^31^. Comparing the manufactured plywood to the traditional phenol–formaldehyde resin, the mechanical characteristics of the former were higher. The tannin addition decreased the toxicity and improved the environmental friendliness of the starch-based wood glue while simultaneously speeding up the reaction time.

Fillers are added in addition to tackifiers to modify starch in order to lower formulation costs, enhance consistency and mechanical properties^32^, increase electrical or thermal conductivity^22^ decrease moisture absorption, and achieve other goals depending on application specifications. Compared to other materials, starch-based adhesives often have poor cohesive energies and weak intermolecular interactions. The strength and longevity of the adhesive can be increased by adding fillers with the proper aspect ratio and particle size. By cross-linking multiple big molecular chains, the active surface of these filler particles can be used to generate a network structure. When one chemical chain experiences stress, cross-linking allows the stress to be transferred and distributed to other molecules^33^. Rodrigues and Menezes^34^ used clays with montmorillonite to strengthen dental adhesive. Small amounts of clay (0.2%) were added to the adhesive, significantly improving its elastic modulus and thermal resistance without compromising the degree of conversions.

In this research, kaolin was used as a filler material due to its unique properties to overcome the problems associated with neat starch based adhesive^35,36^ and tannic acid aslo used as tackifier. This study focuses on utilizing cassava root as a source of starch for adhesive development and characterize the performance enhancemet on the prepared biobased adhesive through physciochemical and mechnical properties.

## Materials and methods

### Materials and chemicals

The cassava root was obtained from Hawassa, southern Ethiopia. The filler material (kaolin) is locally available in south Gondar, particularly in Adiszemen, which is 112 km far away from Bahir-Dar city. Laboratory grade Tannic acid was obtained from the organic laboratory, chemical engineering department. The main equipment for conducting mechanical, chemical and physical properties analysis were: weighing balance (JF-2004), peeler, grinder, sieve, beakers, water bath (TBS451PA) shaker, measuring cylinder, burettes, nylon cloth, magnetic stirrer, heating oven (DHG-9023A 20L), different-sized standard measuring flasks, UV spectrophotometer (UV-2700), tensile tester (UTM 100ST to measure peel, shear and tensile strength.

### Methods

#### Isolation of starch

**Fig. 1:**
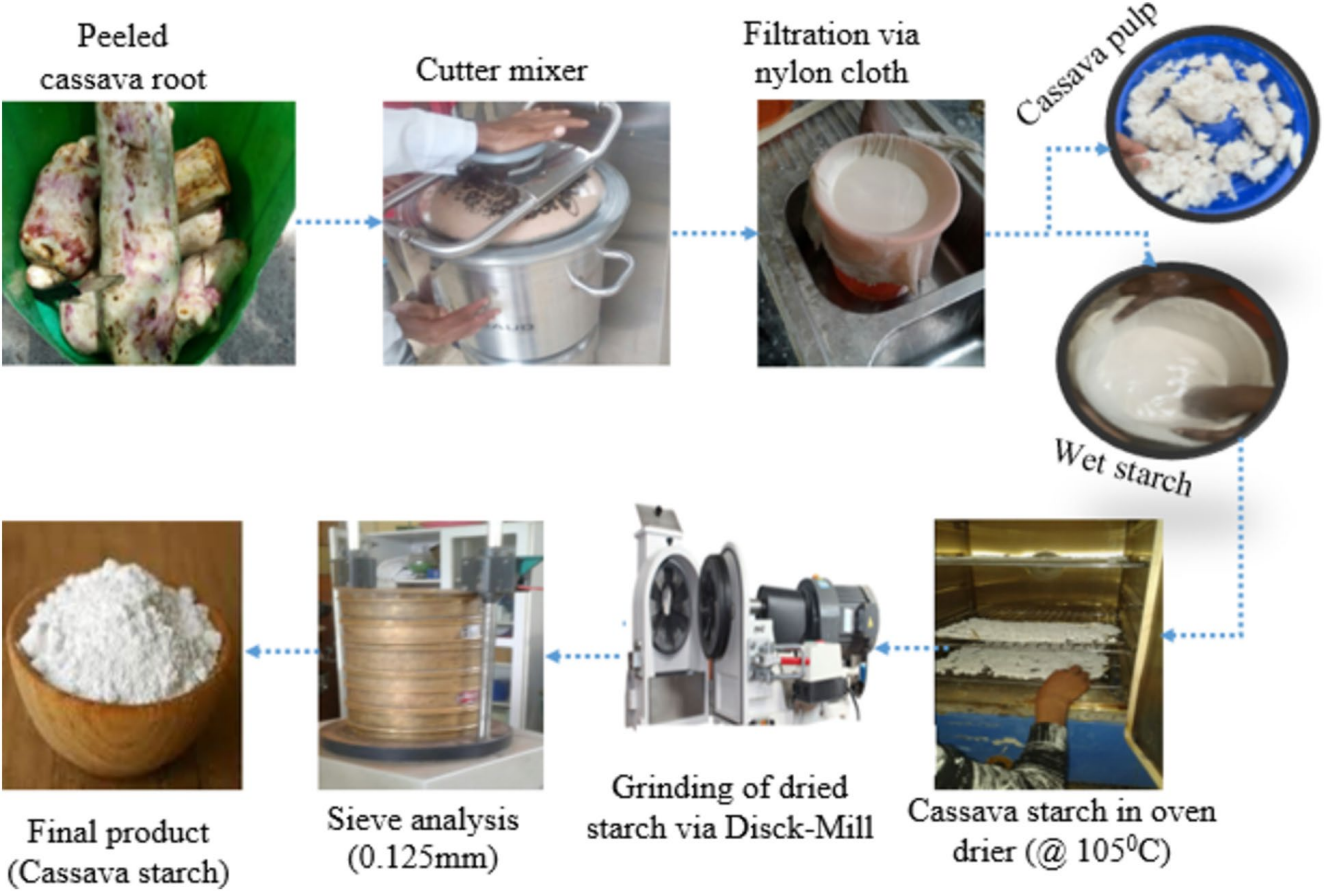
Cassava starch extraction process.

The conventional procedure was used to separate the cassava starch^37^. As shown in Fig. 1, harvested cassava roots were peeled to remove the scaly, brown flesh. After the cassava was peeled, it was thoroughly cleaned and transported to a milling facility where it was ground. To achieve a fine seepage of cassava, the slurry was meticulously filtered, dried, and then selectively milled after the grinding process^38^. After that, the water and starch sediment are separated by letting the seepage stand for 12 h. At the end of the separation, decanting separates water from the starch sediment. The residual wet starch was dried in an oven for six to ten hours at 45 °C^39^.

#### Preparation of hot melt adhesives

**Fig. 2:**
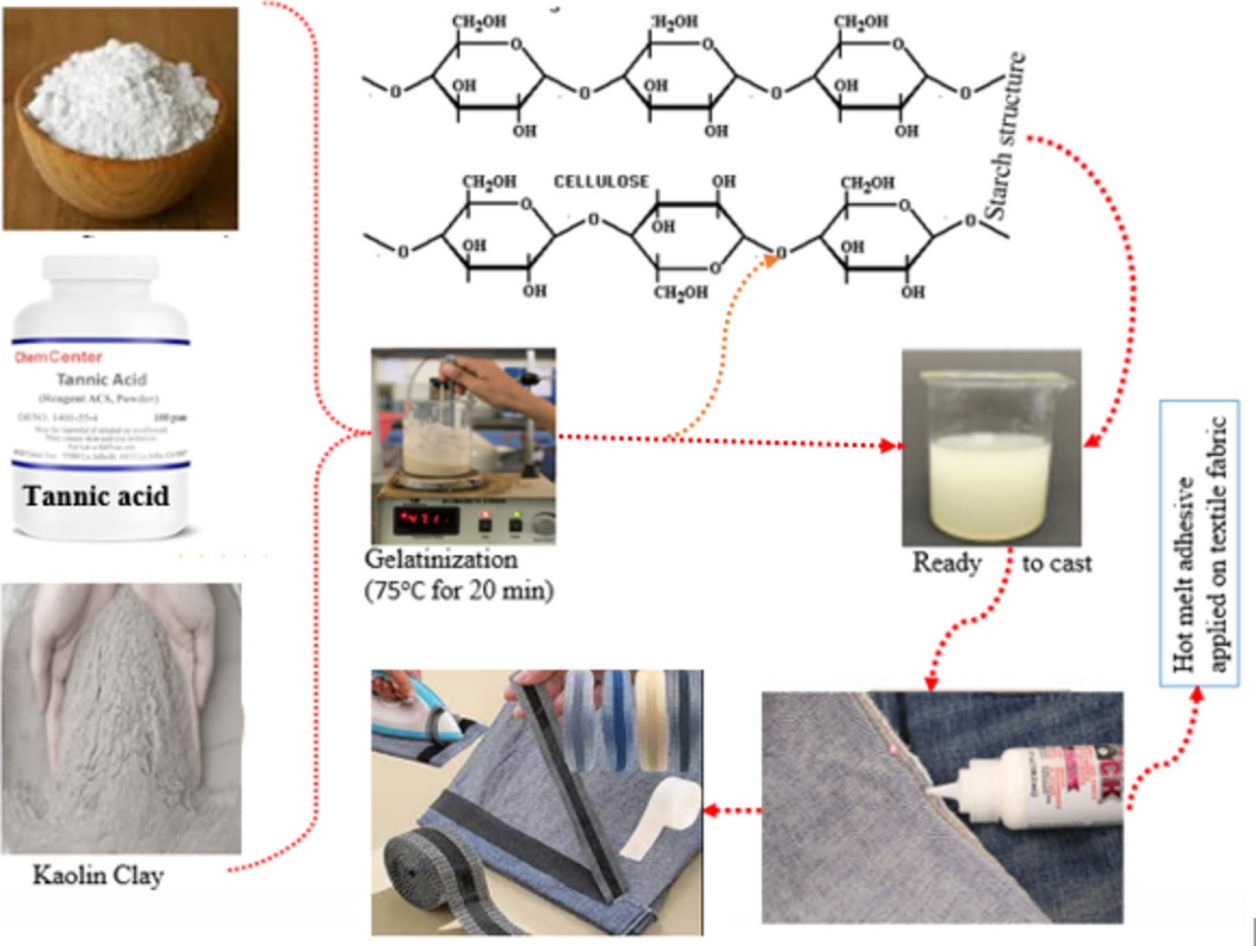
Development of hot melt Adhesive and application on textile fabrics.

The development of adhesive was performed as illustrated in Fig. 2. To make a suspension solution, 5 g of starch was dissolved in one hundred milliliters of distilled water. According to the experimental design, the filler material with 10, 30, and 50% w/w and the takifier with 10, 20, and 30% v/w were added to the hot melt solution and combined. The adhesive-forming solutions were then allowed to reach room temperature before being stored in a plastic container that was sealed for use and additional property testing. On adherends, the adhesive precursor solutions were dispersed and applied. After the adhesive was applied, two adherents were positioned side by side on the benchtop, overlapped, and cemented together. The starch-kaolin-tannic acid formulations and controls were dried and cured at room temperature for one hour (25 °C) before being placed in an oven set at 120 °C for twenty-four hours in order to test the bond strength. The joints were allowed to cool for one hour at room temperature before testing. The starch, kaolin, and tannic acid controls were first cured in typical, dry circumstances before being tested for water resistance. After that, these bonded joints were submerged in deionized water for 24 h at 40 °C. Lap shear testing was carried out right after the samples were removed from the water bath while still wet^1,38^.

#### Physico-chemical characterization of hot melt adhesive

##### Viscosity

The prepared solution was heated at a rate of 10 °C/min in a boiling water bath at several temperature ranges between 50 and 80 °C. The cycle of heating and cooling was employed during the procedure. The gelatinized solution was finally allowed to cool to 50 °C at a rate of 15 °C per minute, and it was maintained there for a minute. A viscometer operating at 95 rpm was used to measure the viscosity^40^. Throughout the adhesive creation process, the impact of temperature at each 10 °C intervals was noted.

##### Solids content (SC)

The solids content of the adhesive was determined using the oven-drying method according to the standard. Approximately three grams of initial weight (W1) of the adhesive was placed into an oven and the temperature was set to 105 °C for 24 h until a constant weight (W2) was obtained. The value of the solids content was calculated using Eq. (1).

1 $$\text{SC }\left(\text{\%}\right)= \frac{\text{w}1}{\text{W}2}*100$$

##### Moisture content (M %)

Moisture of adhesive was determined according to the^41^ method. The material was dried in the oven at a temperature of 105 ± 5 °C for two hours and the sample was cooled down to room temperature prior to determining the moisture content”. Moisture content of the sample then was determined as follows: Mass of water in sample = mass of wet sample—mass of dry sample. Mw-mass of water. Md = mass of dried sample.

2 $$({\text{M}}\% ) = \frac{{{\text{Mw}}}}{{{\text{Md}}}}*100$$

##### Shear strength

The shear strength of the adhesive in terms of (both in wet and dry state) was determined by using the procedures of ASTM D903-49^5,42^. Pieces of clothes, with dimensions of 15 mm × 15 mm × 5 mm were glued with adhesives at room temperature for 24 h. Before shear strength determined, the glued specimens were stored in the laboratory at 23 ± 2 °C and 50 ± 5% humidity for 48 h. The shear strength was calculated as following: M = F_max_/A, where M (MPa) is the shear strength, F_max_ (N) is the observed maximum failing load, and A (mm^2^) is the bonding surface of the sample. The testing speed was 2 mm/min.

##### Tensile strength

The mechanical properties in terms of tensile strength (Mpa) of the adhesive were determined according to ASTM D-882-9 using the universal testing machine (UTM-1422). Samples were cut into 25.4 mm width and 80 mm length, with an overlap length maintained at 30 mm. The wood lap joint specimen was fixed until the adhesive was fully cured at room temperature. The tensile strength was determined using a Universal Testing Machine (UTM) with a crosshead speed of 2 mm/min. The average load required to separate the adherents was measured in kilonewtons per square meter (kN/m^2^)

3 $$\text{TS }\left(\text{\%}\right)= \frac{\text{Force at peak}}{\text{Area of piece subjected to peak}}*100$$

where TS is tensile strength.

### Fourier transform infrared (FTIR)

A Fourier transform infrared spectrophotometer (Jasco-FT/IR-6600A) was used to investgate the adhesive functional surface groups and chemical bonds (interaction forces). Using a mechanical press, the sticky film pieces were combined with spectral grade KBr (1:100) and formed into pellets. With a resolution of 0.4 cm^−1^, the spectra were obtained in the range of 4000–400 cm^−1^ in terms of percent transmittance.

## Results and discussion

### Physico-chemical properties of hot melt adhesive

#### Viscosity

**Fig. 3:**
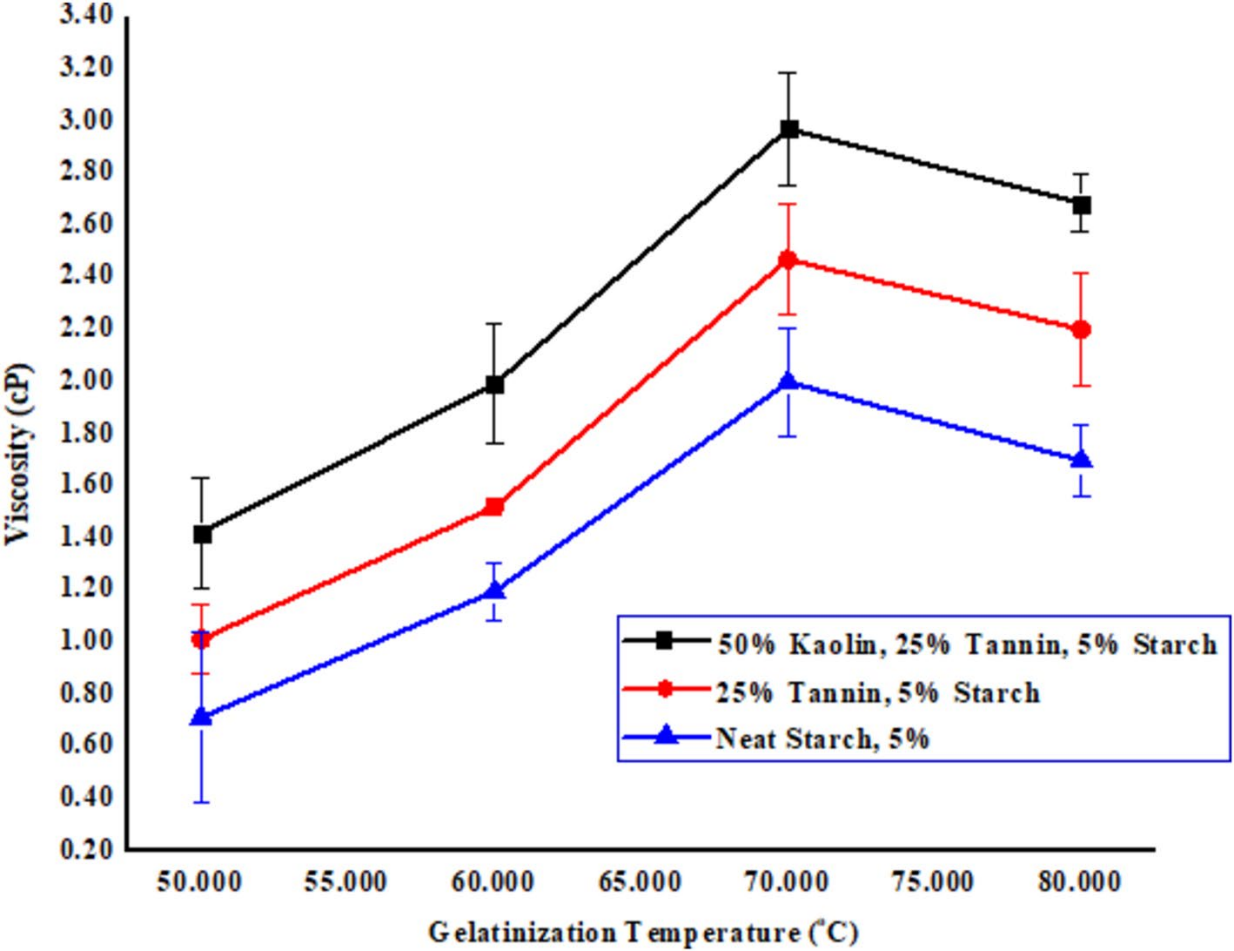
Effect of temperature on the viscosity of adhesive.

Viscosity highly correlated with the adhesive properties^27^. The adhesive surface tension must be less than/equal to the surface energy of the material to achieve good molecular interaction.^43^ The gelatinization process was achieved by heating–cooling system with water^44^. Kaolin have a tendency to increase the viscosity^45^. As shown in Fig. 3 from the beginning, viscosity was slightly increased in all three cases (kaolin + tannin + starch, tannin + starch, Neat starch)^46^. This results from a small amount of amylose molecules diffusing out of the starch granules, causing the granules to expand. Amylose has relatively weaker hydrogen bonds compared to amylopectin, allowing it to more easily migrate out of the granular structure^14^. From a 50–60 °C in the first graph line, there is only a small increment of viscosity from 1.42 ± 0.21 to 1.99 ± 0.23 cP (Kaolin + Tannin + starch), in the second graph line (Tannin + starch) 1.012 ± 0.13 to 1.519 ± 0.03 cP. In the third graph line, the viscosity was decreased beyond 75 °C from 0.712 ± 0.33 to 1.195 ± 0.11. This is due to the kinetic energy of starch molecules almost they are negligible implied that starch molecules have high stored energy/modulus due to the formation of strong hydrogen bonding among the ingredients^47^. As shown in Fig. 3, in the second stage for all three graph lines, from a temperature interval of 60–70 °C, the viscosity was tightly increased for (Kaolin + Tannin + starch) from 1.99 ± 0.23 to 2.968 ± 0.22 cp, for the second graph line (Tannin + starch) 1.519 ± 0.03 to 2.468 ± 0.21cP. For the third graph line 1.195 ± 0.11 to 1.998 ± 0.21 cp This is due to the intermolecular interaction effect of extra granular (amylose and amylopectin) material, large amounts of amylose leached out and swelling of amylopectin granule started^48^. In the end for three-graph lines cases from a temperature interval of 70–80 °C, the viscosity of the respective graph lines were found to be 2.968 ± 0.22 to 2.684 ± 0.11 cp, 2.468 ± 0.21 to 2.199 ± 0.22 cp and 1.998 ± 0.21 to 1.696 ± 0.14 cp. The maximum viscosity was found to at a temperature interval of (Kaolin + Tannin + starch) which is 2.968 ± 0.22 cp^46^.

#### Solid content

**Fig. 4:**
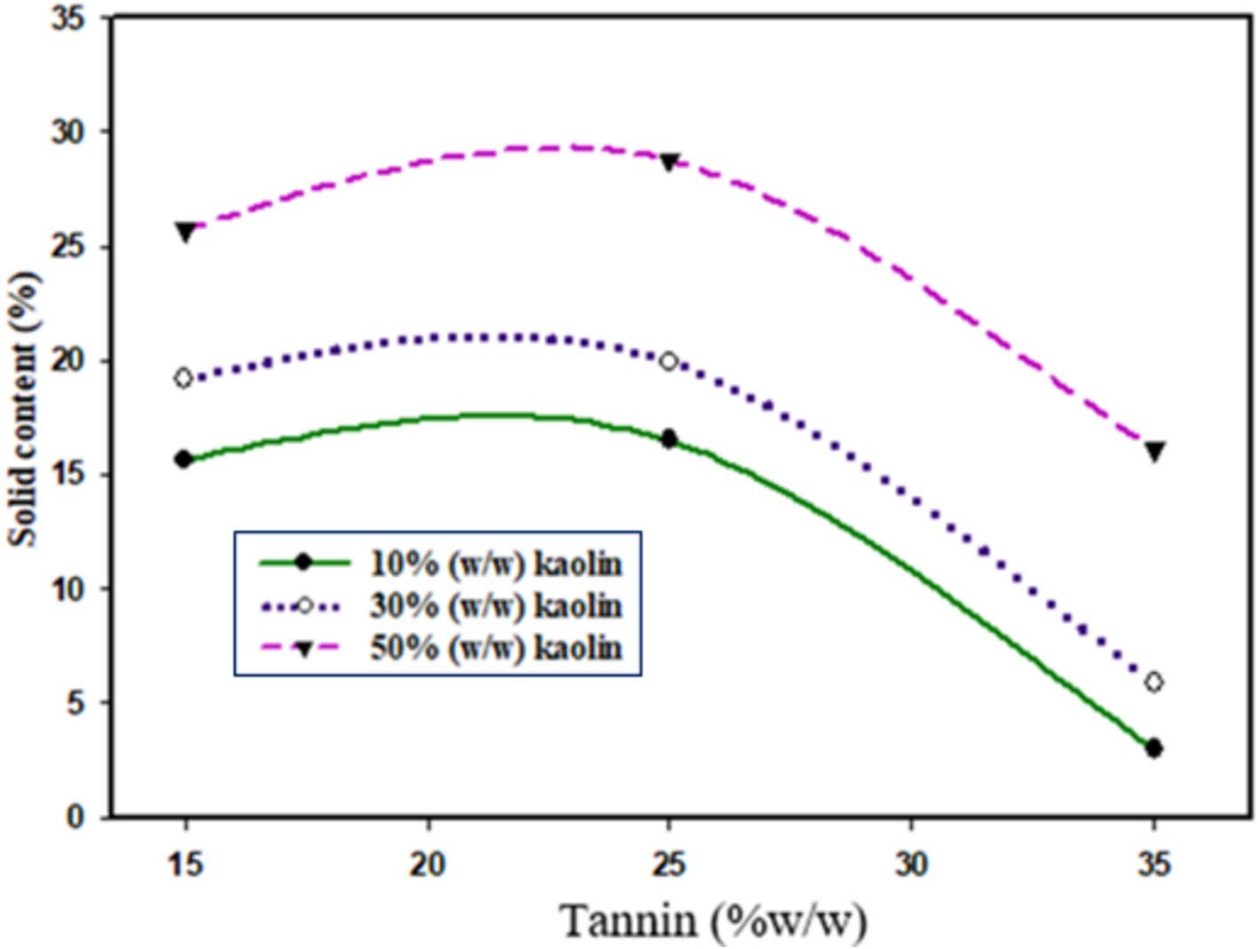
Effect of kaolin and tannin on the solid content of adhesive.

The data presented in Fig. 4**,** demonstrates that the solid content of the adhesive material increased from 15.63 ± 0.1% to 19.18 ± 0.12% as the kaolin content is raised from 10 to 30% w/w, and 19.18 ± 0.12% to 25.72 ± 0.22% with a tannin content of 15%. The addition of kaolin is not only responsible for to increase the solid content but also to form a good network among all the ingredients (tannin, starch). Generally, the increase in kaolin content results higher solid content. Therefore, the optimum value was found to be 28.77 ± 0.12% (50% kaolin with 25% tannin)^49^. The relationship between kaolin and solid content directly correlated with the curing rate, in terms of temperature and time. Specifically, the solid content increased as the curing rate become elevated, and this led to a shorter observed gel time when the solid content was lower^50^. The incorporation of kaolin also responsible for enhancing the solid content^51^. Here also the adherends were too tighter and more compact structure in the surface layers^52^. The addition of the kaolin into an a starch reduces its moisture uptake, and thus helps to increase its solid content^52,53^

### Moisture content

**Fig. 5:**
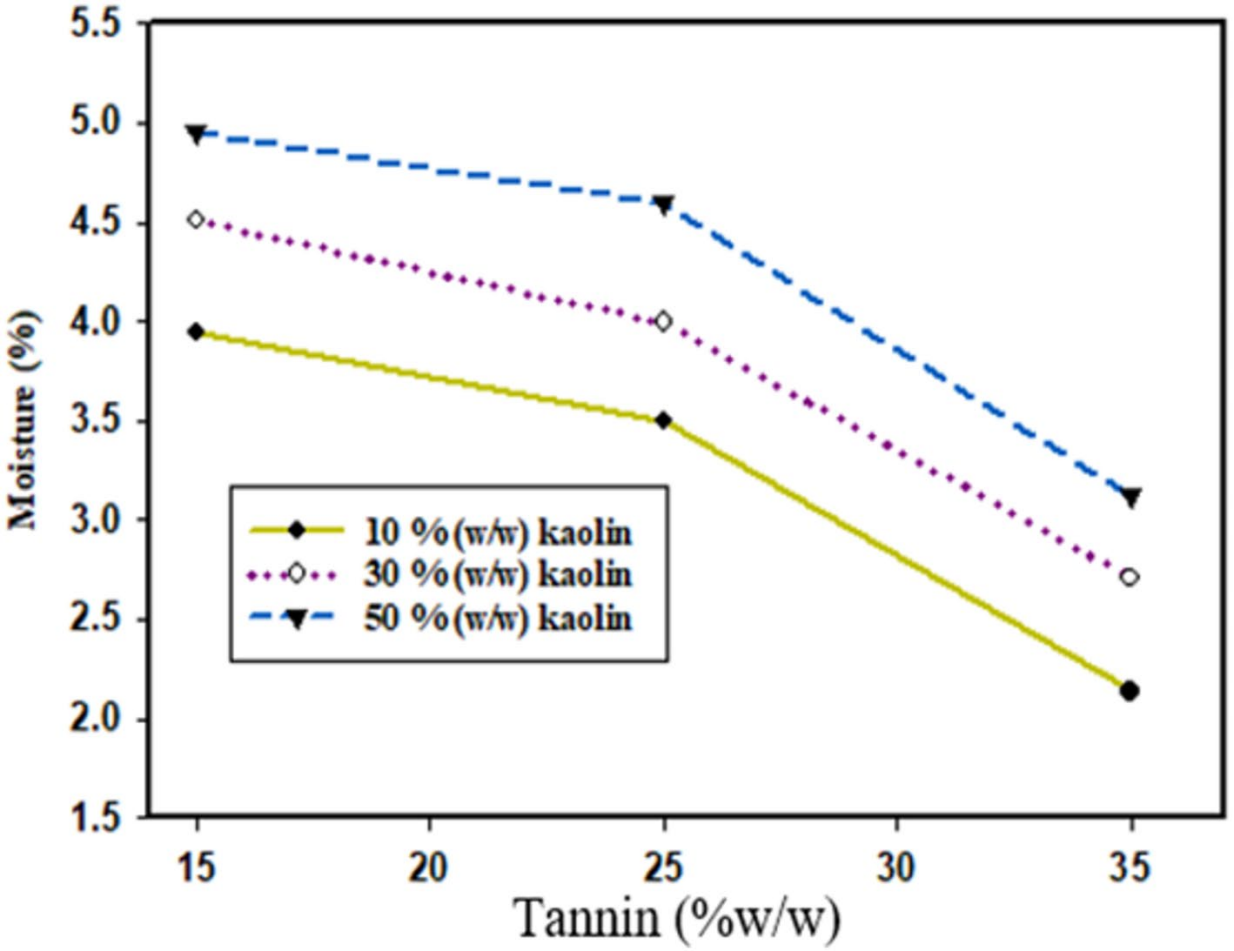
Effect of kaolin and tannin on the moisture uptake of adhesive.

Based on Fig. 5**,** as shown below, from the beginning when the kaolin content increases from 10 to 30%, the moisture uptake of the adhesive a little bit increased from 3.94 ± 0.12 to 4.51 ± 0.22%**.** This is because of kaolin has the tendency absorb moisture^14^. On the other side, when the tannin concentration increased from 15 to 25%, the moisture uptake was decreased from 4.95 ± 0.11 to 3.49 ± 0.22percentage. According to^7^ findings, the moisture content of dextrin starch based adhesive was found to be 58% which is higher than the present study which implied that the present results are good in terms of shelf life and moisture resistance. Up to 25%, the moisture uptake by hot melt adhesive was slightly decreased but beyond 25% of tannin, the moisture uptake by hot melt adhesive was sharply decreased this is because of the formation of strong cross linking agent between the starch and tannic acid^27^.

### Shear strength

**Fig. 6:**
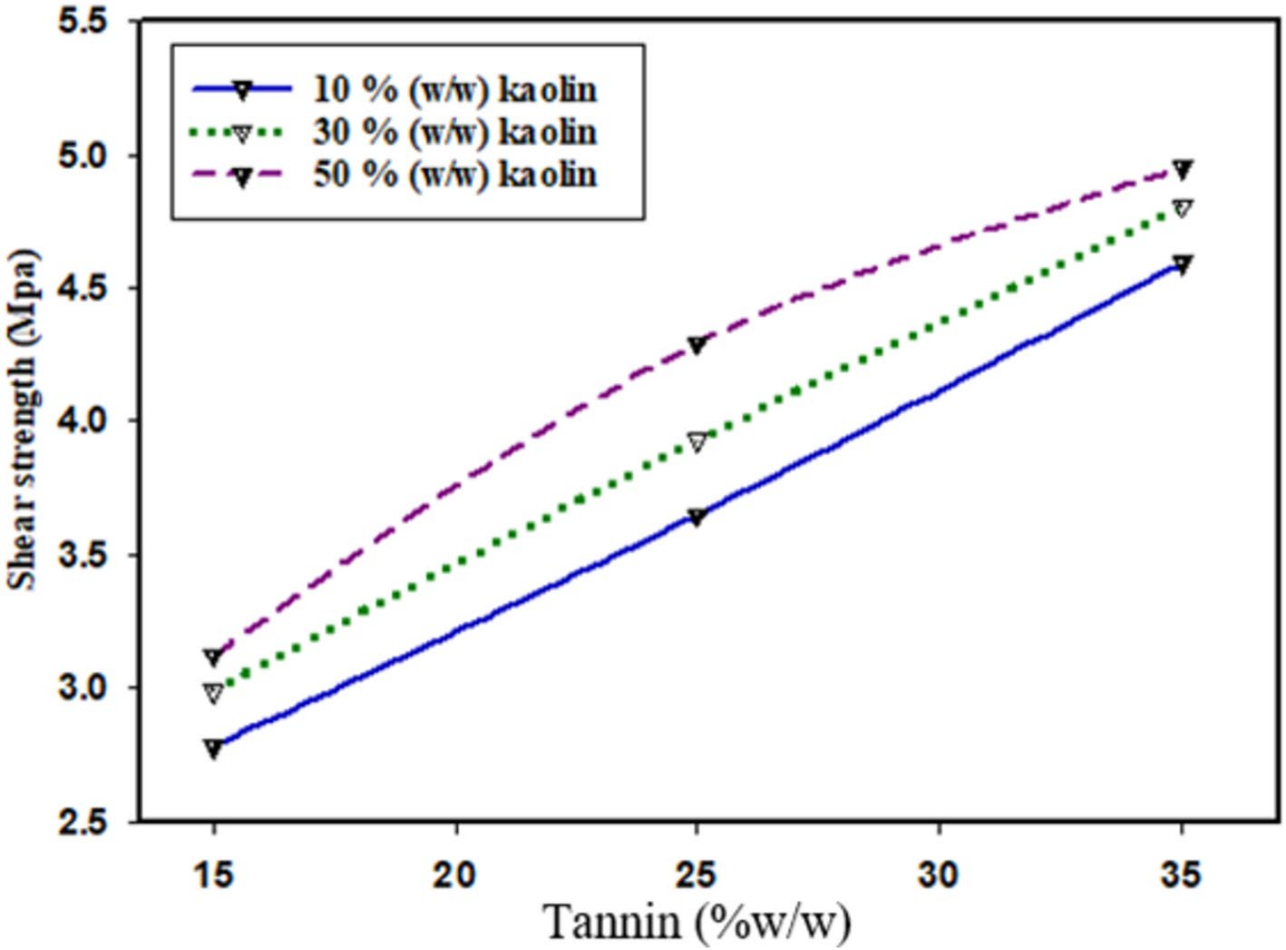
Effect of kaolin and tannin on the shear strength of adhesive.

Shear strength means the measurement of deformation under constant shear stress, & related to the internal or cohesive strength of the product (adhesive) material^54^. It is a highly essential parameter reflects the bonding ability of the adhesive material^55^. This used to estimate the water resistance of the adhesive in wet state^56^. According to Zhang et al.^57^ studies, the optimal limit of shear strength for starch based adhesive was 4.3 Mpa and 2.17 MPa in the dry and wet state, respectively. Based on the present experimental result as shown in Fig. 6**,** the maximum shear strength of the adhesive in the dry state was found to be 4.93 ± 0.11while the shear strength in the wet state was 2.15 ± 0.22 Mpa. The presence of kaolin and tannin significantly improved the bonding strength, especially the shear strength at high curing temperatures^54,55^. Tannin has a great impact on the on the shear strength of the adhesive^27^. According to the findings of, tackifier materials are added to adhesive formulations to improve the tack, adhesion, mechanical properties (shear, tensile strength, peel strength), and viscoelastic behavior of the overall adhesive system.

The addition of tackifiers reduces the viscosity of the adhesive, which facilitates increased molecular contact between the surfaces being adhered. Tackifiers also improve molecular contact by enabling the adhesive to flow more easily into the surface of the adherend. The inclusion of tackifiers (tannins) enhances the green strength or cohesive strength of the adhesive, allowing the bonded joints to better resist separation. The increased flow, enhanced molecular contact, reduced viscosity, and improved cohesive strength provided by the tackifiers. This combination of tackifier-induced improvements to wetting, viscosity, and cohesive properties appears to be critical for the observed enhancement in the peel performance of these adhesive systems^59^.

### Tensile strength

**Fig. 7:**
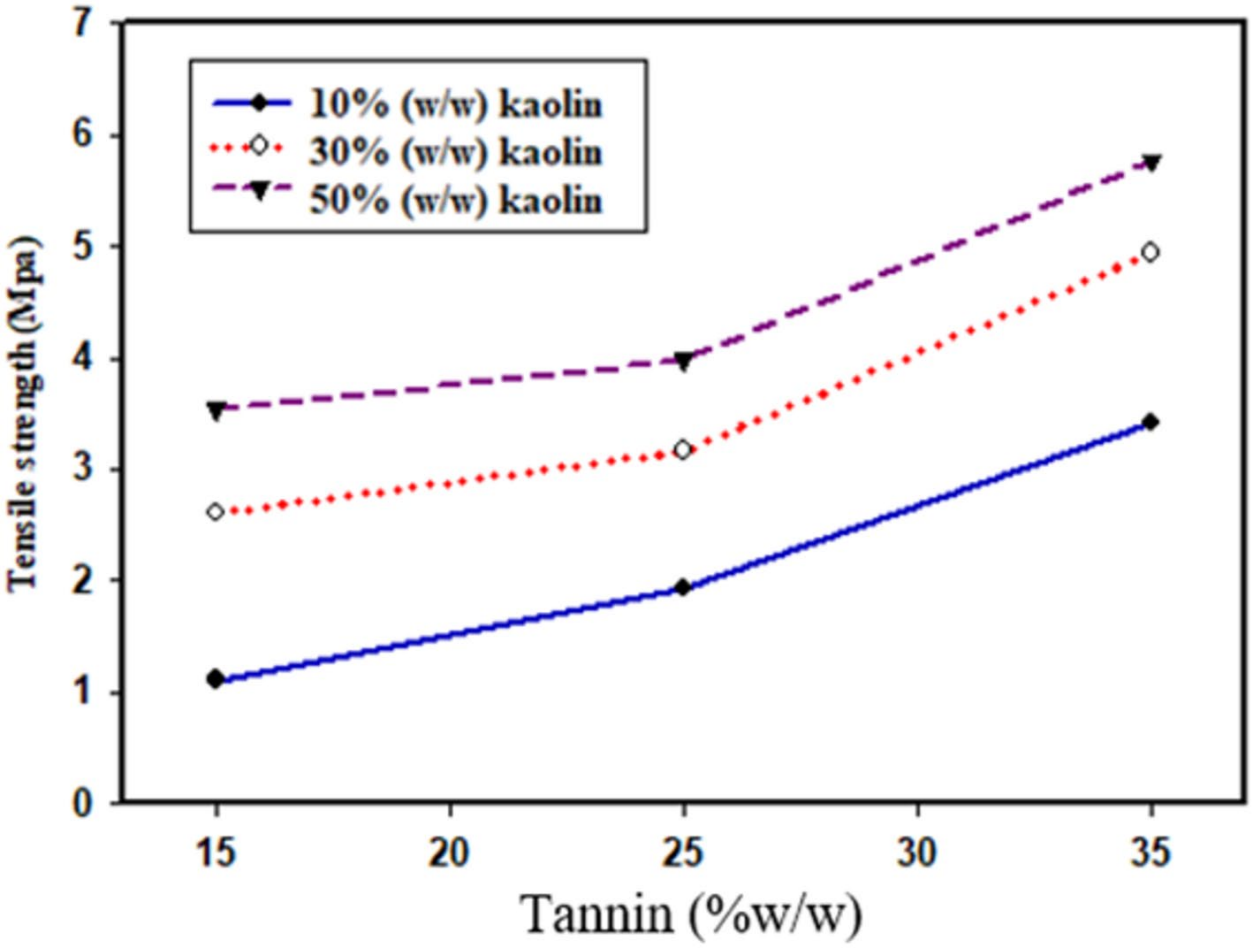
Effect of kaolin and tannin on the tensile strength of adhesive.

The cohesive strength of the adhesive was studied using universal tensile test^60^. As shown in Fig. 7, The addition of tannic acid (15–35%) provided that cohesive strength to adhesive properties^1^. The tensile strength was carried out to determine the force required to pull apart the bonded substrates and to measure the adhesive bond’s response to the applied stress^61^. The optimum tensile strength of the adhesive as shown in Fig. 7**,** was found to be 5.76 ± 0.03 Mpa, which found at 35% tannin concentration and 50% kaolin concentration. The minimum tensile strength of adhesive in a dry state was obtained 1.11 ± 0.12 Mpa at 10% kaolin concentration and 15% tannin content^27^. This differs from the peel test, as the act of peeling and pulling are different but the two tests focus on bringing out the adhesive that provides the strongest bonds^62^**.** The strong adhesion strength (5.76 ± 0.03 Mpa), is likely related to the extended structure of tannic acid^1^. The bio-based adhesives also showed that an increase in mechanical strength which exhibited favorable thermal stability^43^. This is due to movement from polymer chain which causes water molecules, it impacted the amylose and amylopectin from strong hydrogen bonding, so it happens recrystallization or retrogradation^63^. Past studies had reported that for a miscible ethylene–vinyl acetate (EVA)/tackifier system, the temperature at which the maximum adhesive tensile strength was observed tended to shift towards higher temperatures as the tackifier content of the blends increased^59^.

### FTIR analysis

**Fig. 8:**
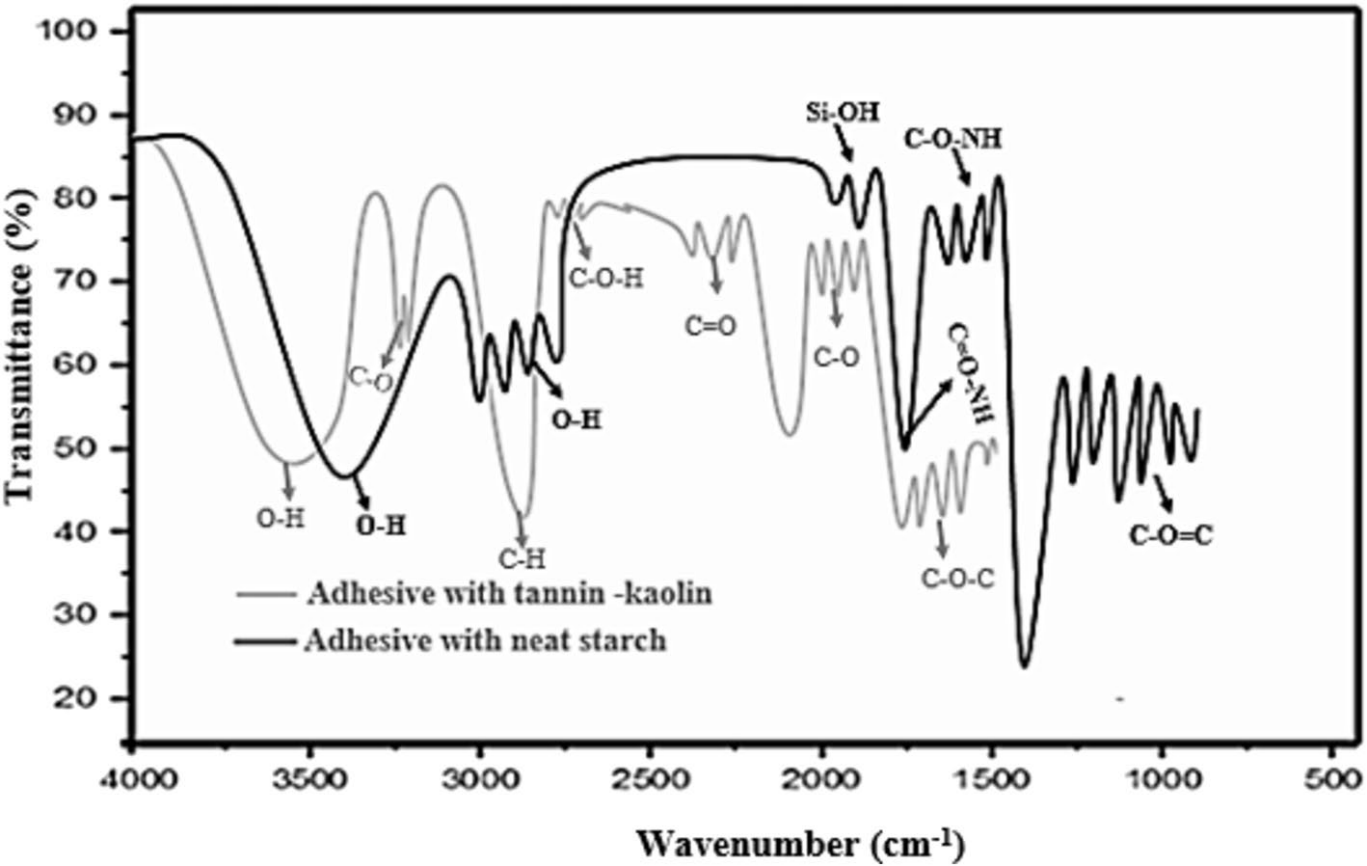
FITR analysis for adhesive with tannin and kaolin (light black) and Neat starch (dark black).

An infrared spectrum is a plot of percent transmittance or absorbance against wave number^55^. FTIR analysis was performed to clarify the possible formation of chemical interactions among the parameters in the adhesive^64^. The analysis typically identified the possible molecular interactions of constraints such as starch, filler, and tannin, as shown in, Fig. 8**,** both adhesive samples (one with filler and the other without filler) displays characteristic peaks as shown in the figure. The best of constraints interaction (filler, tannin and starch) were observed at the absorption band around 3100 cm^−1^, and 1250–1100 cm^−1^^65^. The larger wavenumbers (shorter wavelengths) are associated with higher frequencies and higher energy. The O–H polar bond mostly showed that strong and broad absorption bands that are easy to identify. The broad shape of the spike (absorption band) results from the strong hydrogen bonding of -OH groups between each molecules^66^. The spectra indicated that a broad absorption band at 3274 cm^-1^ for O–H stretching vibrations and a smaller absorption band at 1150 cm^-1^ attributed to C–H stretching vibration. At 1150, 1000 and 890 cm^-1^ wavelengths describe C–O–C stretching and of all the starch samples is an evidence for the vibration of the glycosidic linkage. Water molecule is corresponding to a band at a wave number of 1700 cm^-1^^66^. Strong peak shown for –OH groups in the starch granules, correspond to moderate level of moisture content, which is important in making good bio based adhesive. High amount of -OH bonding gives more availability of starch chains to be hydrolyze resulting an increase of viscosity of the adhesive. Thus, the addition of filler materials strengthen the bonding and thickening ability of the bio-based adhesive^67^. The product showed good interaction, which mainly resulted from increased number of hydroxyl groups, attached to the starch as well as from gelatinization that had broken the inter/intra-molecular bond.

## Conclusion

Based on the present study, it was demonstrated that starch-based hot melt adhesives can be successfully produced from cassava root starch. The developed starch-based hot melt adhesive exhibited excellent thermo-mechanical performance, moisture uptake resistance, bonding strength, and stability. The key findings of this study indicate that the starch-based hot melt adhesive showed good thermal stability and mechanical properties, making it suitable for textile industry applications. The adhesive also demonstrated superior resistance to moisture absorption, which is a crucial requirement for textile applications where the adhesive is exposed to various environmental conditions. Furthermore, the starch-based adhesive exhibited excellent bonding strength, ensuring reliable and durable textile assemblies, and displayed good shelf-life stability, maintaining its performance characteristics over an extended period. The incorporation of kaolin and tannin as reinforcing agents further enhanced the mechanical properties and matrix formation of the starch-based adhesive, as evidenced by the FTIR analysis. In conclusion, the findings of the present study suggest that the developed starch-based hot melt adhesive, derived from cassava root starch and reinforced with kaolin and tannin, can be a viable and sustainable alternative to conventional adhesives in the textile industry. The adhesive’s excellent performance characteristics, coupled with its eco-friendly nature, make it a promising solution for various textile fabric bonding applications.

## Data Availability

All the necessary data is avaliable in the manuscript.
